# Network pharmacology and experimental verification to decode the action of Qing Fei Hua Xian Decotion against pulmonary fibrosis

**DOI:** 10.1371/journal.pone.0305903

**Published:** 2024-06-24

**Authors:** Hao-Liang Ke, Rui-Jie Li, Chao-Chao Yu, Xiu-Ping Wang, Chao-Yan Wu, Ying-Wen Zhang

**Affiliations:** 1 Department of Integrated Chinese and Western Medicine, Zhongnan Hospital of Wuhan University, Wuhan, Hubei, China; 2 School of Traditional Chinese Medicine, Hubei University of Chinese Medicine, Wuhan, Hubei, China; 3 Department of Rehabilitation, Union Hospital, Tongji Medical College, Huazhong University of Science and Technology, Wuhan, Hubei, China; National Institutes of Health, UNITED STATES

## Abstract

**Background:**

Pulmonary fibrosis (PF) is a common interstitial pneumonia disease, also occurred in post-COVID-19 survivors. The mechanism underlying the anti-PF effect of Qing Fei Hua Xian Decotion (QFHXD), a traditional Chinese medicine formula applied for treating PF in COVID-19 survivors, is unclear. This study aimed to uncover the mechanisms related to the anti-PF effect of QFHXD through analysis of network pharmacology and experimental verification.

**Methods:**

The candidate chemical compounds of QFHXD and its putative targets for treating PF were achieved from public databases, thereby we established the corresponding “herb-compound-target” network of QFHXD. The protein–protein interaction network of potential targets was also constructed to screen the core targets. Furthermore, Gene ontology (GO) and Kyoto Encyclopedia of Genes and Genomes (KEGG) pathway enrichment analysis were used to predict targets, and pathways, then validated by in vivo experiments.

**Results:**

A total of 188 active compounds in QFHXD and 50 target genes were identified from databases. The key therapeutic targets of QFHXD, such as PI3K/Akt, IL-6, TNF, IL-1β, STAT3, MMP-9, and TGF-β1 were identified by KEGG and GO analysis. Anti-PF effects of QFHXD (in a dose-dependent manner) and prednisone were confirmed by HE, Masson staining, and Sirius red staining as well as in vivo Micro-CT and immunohistochemical analysis in a rat model of bleomycin-induced PF. Besides, QFXHD remarkably inhibits the activity of PI3K/Akt/NF-κB and TGF-β1/Smad2/3.

**Conclusions:**

QFXHD significantly attenuated bleomycin-induced PF via inhibiting inflammation and epithelial-mesenchymal transition. PI3K/Akt/NF-κB and TGF-β1/Smad2/3 pathways might be the potential therapeutic effects of QFHXD for treating PF.

## Introduction

Pulmonary fibrosis is a common lung disease, characterized by a chronic, progressive, and irreversible interstitial disease process. The development of PF involves many cellular pathological processes, including alveolar epithelial cell apoptosis, infiltration of inflammatory cells, epithelial-mesenchymal transition (EMT), extracellular matrix remodeling, fibroblast proliferation, and differentiation into myofibroblast, etc., thereby leading to the lung tissue scar formation, distortion of the alveolar architecture, and inexorably declines of lung function. It has been reported that the average survival time of PF is 3–5 years after the diagnosis [1]. Due to the COVID-19 pandemic spread worldwide, the COVID-19 patients have an increased risk of fibrotic lung disease, thus the potential global burden of fibrotic lung disease in the future will probably be continuing to increase considerably [2]. However, the FDA-approved drugs for treating PF, such as pirfenidone and nintedanib [3], still remain a great controversial for their severe adverse effects and limited improvements in survival or quality of life [4–6]. To this end, it is necessary to develop the potent anti-fibrotic therapies.

Among the multicellular pathological processes in PF, the involvement of epithelial-mesenchymal transition (EMT) is critical for PF development. When EMT occurs in the lung, there were decreased E-cadherin in epithelial cells as well as increased α-smooth muscle actin (α-SMA), and N-cadherin in mesenchymal cells [7]. In addition, approximately one‐third of fibroblasts were considered originating from epithelial cells in pulmonary fibrosis [8]. Myofibroblasts derived from epithelial cells through EMT have dysregulated proliferation in excess of extracellular matrix (ECM) [9, 10]. Besides, inflammation and the immune reactions in response to inflammation also contribute to the pathogenesis of PF, such as infiltration of inflammatory cells and production of pro-inflammatory cytokines, all of which promote PF development through injury of both alveolar epithelial cells and capillary endothelial cells [11, 12]. As reported, inhibition of certain signaling pathways can ameliorate bleomycin-induced pulmonary fibrosis, such as NF-κB/NLRP3 signaling-mediated inflammation [13], the IL-1β/IL-1R1/MyD88/NF-κB axis [14], and IL-6/STAT3 pathway [15]. Therefore, targeting EMT and inflammatory response could be potential approaches to prevent PF development.

According to the theories of traditional Chinese medicine and clinical experience, Qing Fei Hua Xian Decotion (QFHXD) has been applied in clinic for treating PF in COVID-19 survivors, with a satisfactory treatment efficacy in alleviating clinical symptoms and receding the disease progression. The content of QFHXD includes 14 medicinal herbs as follows: *Astragalus membranaceus*, *Angelica sinensis*, *Ephedra sinica Stapf*, *Prunus armeniaca*, *Pinellia ternata*, *Trichosanthes kirilowii Maxim*, *Pseudostellaria heterophylla*, *Lepidium apetalum Willd*, *Paeonia lactiflora Pall*, *Areca catechu L*, *Citrus aurantium L*, *Fritillaria thunbergii Miq*, *Luffa cylindrica Roem*, *and Radix Glycyrrhizae*. Many of these herbs or their bioactive components are proved to have significant anti-fibrosis effects. For example, the Astragaloside IV extracted from astragalus membranaceus can reverse EMT in the animal model of bleomycin-induced PF via inhibition of FOXO3a hyperphosphorylation induced by TGF-β1/PI3K/Akt [16]. In addition, Astragaloside IV also ameliorated renal interstitial fibrosis through suppression of TLR4/NF-кB-mediated inflammation [17]. It was also reported that angelica sinensis polysaccharide can alleviate chronic liver fibrosis via inactivation of hepatic stellate cells by targeting the IL-22/STAT3 pathway [18]. Therefore, it is reasonably assumed that anti-EMT and anti-inflammation effects of QFHXD are essential for the mechanisms related to its anti-PF function, which should be confirmed through experimental studies.

It is widely accepted that Chinese herbal medicines have multiple active components and multiple targets. Network pharmacology is a useful research tool to explore the interaction between the multi-target drugs and the multiple biological processes in the body, based on the perspectives of systematic pharmacology, which may to some extent share with the principles of the holistic view of Chinese medicine [19]. Thus, the method of network pharmacology may be used to uncover the complex mechanisms of Chinese herbal medicine. In the present study, the network pharmacology method was used for identification of the potential targets of QFHXD for treating PF. The identified key targets were enriched and further verified in a rat model of bleomycin-induced lung fibrosis. Our study brings a novel insight into the pharmacological mechanism of anti-PF effects of QFHXD, which might be informative for develop novel intervention for treating pulmonary fibrosis.

## Materials and methods

### Part I. Network pharmacology-based analysis

#### 1. Identification of candidate compounds in QFHXD

The constituents of QFHXD were searched using TCMSP, (the traditional Chinese medicine systems pharmacology database and its link is http://tcmspw.com. The druggability of each constituent gained from TCMSP was examined, according to its oral bioavailability (OB) and drug-likeness (DL). OB is defined as the absorptivity and bioactivity of the active drug ingredient or active moiety derived from the drug. The index of DL is referred to the pharmacokinetic properties of the druggable constituents, such as their chemical stability and solubility. When its DL index ≥0.18, the drug ingredient was considered high druggability. The higher score of OB and DL index it has (OB ≥ 30% and DL ≥ 0.18), the more opportunity for the constituent to be recognized as a candidate compound. Based on the criteria as recommended by TCMSP, the constituents of QFHXD with OB ≥ 30% and DL index ≥0.18 were selected as active substances in our study. For those that cannot be achieved from TCMSP, such as Loofah Sponge, the Pubchem platform (https://pubchem.ncbi.nlm.nih.gov/) was used to obtain their definite structures. The possible protein targets of the candidate compound were predicted via the Web SwissTargetPrediction [20] (http://www.swisstargetprediction.ch/).

#### 2. Identification of gene targets related to pulmonary fibrosis(PF)

Protein targets related to pulmonary fibrosis were identified from four databases, including Disgenet (https://disgenetplus.com/), Drugbank (https://go.drugbank.com/), OMIM (https://www.omim.org/), and TTD (http://db.idrblab.net/ttd/). When looking for the protein targets, only those in “homo sapiens” were considered. Also, the coding genes for protein targets could be collected from UniProt (https://www.uniprot.org/). Furthermore, the same procedure was performed for the identification of the targets of the active compound derived from QFHXD.

#### 3. Conducting PPI network

The protein-protein interactions (PPI) of protein targets related to the active compound derived from QFHXD were analyzed using STRING (Version 11.5, https://string-db.org/), in which the protein-protein associations were denoted by network nodes and edges. Furthermore, Cytoscape software (version 3.7.2) was used to establish two interactive networks of PPI for evaluating QFHXD components and PF-related targets. A core PPI network was finally established by analysis of the opological features of target proteins in the merged PPI network.

#### 4. Gene ontology and pathway enrichment analysis for PF-related targets of QFHXD

The GO enrichment analysis of the candidate target proteins that were obtained from the merged PPI network was performed using DAVID Bioinformatics Resources 6.8 (https://david.ncifcrf.gov/), followed by Kyoto Encyclopedia of Genes and Genomes (KEGG) pathway enrichment analysis to explore the target proteins-related biological pathways. The enriched GO terms were identified by the hypergeometric test. The analysis results were visualized by a bubble chart and a three-in-one histogram via the Bioinformatics platform (http://www.bioinformatics.com.cn/).

#### 5. Construction of networks and analysis

The QFHXD compound-target and the target-pathway networks were generated by software Cytoscape 3.7.2 for exploring the molecular mechanisms underlying the effect of QFHXD on PF. The network nodes donated the compounds, targets, and pathways, while edges for the compound-target and target-pathway interactions in the graphical networks.

### Part II. Experimental validation

#### 6. Herbal medicines and chemicals

Qing Fei Hua Xian Decotion(QFHXD) was prepared by the Jing Brand Chizhengtang Pharmaceutical Co. Ltd.(Hubei, China). QFHXD consists of 14 medicinal herbs, including *Angelica sinensis* (Dang Gui in Chinese), *Astragalus membranaceus* (Huang Qi in Chinese), *Ephedra sinica Stapf* (Ma Huang in Chinese), *Prunus armeniaca* (Ku Xing Ren in Chinese), *Pinellia ternate* (Ban Xia in Chinese), *Trichosanthes kirilowii Maxim* (Gua Lou in Chinese), *Pseudostellaria heterophylla* (Tai Zi Shen in Chinese), *Lepidium apetalum Willd*(Ting Li Zi in Chinese), *Paeonia lactiflora Pall* (Chi Shao in Chinese), *Areca catechu L* (Bin Lang in Chinese), *Citrus aurantium L*(Zhi Shi in Chinese), *Fritillaria thunbergii Miq* (Zhe Bei Mu in Chinese), *Luffa cylindrica Roem* (Si Gua Luo in Chinese), and *Licorice* (Gan Cao in Chinese). All herbs were prepared as dry extracts through water extraction. Bleomycin and Prednisone tablets were obtained from Yeasen Biotechnology (Shanghai, China) Co., Ltd. (lot:Y0125051) and Zhejiang Xianju Pharmaceutical Co. Ltd. (Zhejiang, China), respectively.

#### 7. Animals

Specific pathogen-free adult male Sprague-Dawley rats, aged two months and weighing approximately 200–220 g (laboratory animal quality certification: No. 430727211102922634) were obtained from Hunan SJA Laboratory Animal Co., Ltd (license NO.SCXK(X) 2019–0004). Rats were housed in a room that was kept on a 12 hr light/dark cycle using standard fluorescent lighting, room temperature of 20°C, and relative humidity of 30–60%. Rats were euthanized by carbon dioxide (CO2)-induced asphyxiation, and all efforts were made to minimize animal stress. Our animal study, approved by the Experimental Animal Welfare Ethics Committee, Zhongnan Hospital of Wuhan University (Approval NO. ZN2021068) was performed following the Guide for the Care and Use of Laboratory Animals of the National Institutes of Health (NIH Publications No. 8023, revised 1978). All animal experiments complied with the Policy on the Care and Use of Laboratory Animals of Wuhan University.

#### 8. BLM-induced pulmonary fibrosis in rats and drug administration

All experimental animals were randomly divided into six groups (10 rats per group) as follows: (1) control (normal), (2) BLM (model), (3) BLM + 413.3mg/kg QFHXD g (QFHXD-L), (4) BLM + 826.6mg/kg QFHXD (QFHXD-M), (5) BLM+1239.6mg/kg QFHXD (QFHXD-H), (6) BLM + 0.25mg/kg prednisone (prednisone). The animal equivalent dose (AED) was determined using the following formula: AED (mg/kg) = Human dose (mg/kg) × K_m_ ratio (6.2) [21]. For prednisone, the dose in humans (adult) is 10 mg/60 kg, therefore the AED in rat is approximately 1 mg/kg. For QFHXD, the dose in humans (adult) is 4000mg/60 kg, so the AED in rat equals to approximately 413.3 mg/kg (low-dose), 826.6mg/kg (medium-dose), and 1239.6mg/kg (high-dose). To establish the animal model of PH, rats were anesthetized by inhalation of 1.5% isoflurane, followed by intratracheal injection of BLM (5 mg/kg) in sterile 0.9% saline. From day 1 after intratracheal injection, rats in the control group or model group received intragastrical administration of saline (same volume of experimental drug) for consecutive 28 days. Meanwhile, QFHXD or prednisone was orally administered daily in the corresponding groups for consecutive 28 days. All experimental rats were euthanized at 12 hrs after complement of the micro-CT analysis and the intragastrical injection of BLM or prednisone or saline on the 28^th^ day. Then, their lung tissues were rapidly removed, fixed in 4% paraformaldehyde (partial left lung) for HE, MT, Sirius red staining and immunohistochemical analysis. Other left lungs were weighed (wet weight). Partial tissue of the right lung was harvested and stored at -80°C for subsequent western blotting analysis.

#### 9. In vivo micro-CT analysis

To observe the BLM-induced or drug-induced changes in X-ray absorption, in vivo micro-CT analysis of the whole lung was performed 28 days after drug administration. Animals were anesthetized with 1.5% isoflurane and fixed in the prone position for micro-CT analysis. The CT images were acquired in a micro-CT system (Bruker microCT SkyScan 1276, Germany) with cardiac gating (without respiratory gating), using the following parameters: 85kV; 200μA; image pixel size: 20.258309 μm, and total scan duration of 12–15 minutes. The acquired images were reconstructed with NRecon program (version: 1.7.4.2). The fibrosis degree was used as an indicator of lung fibrosis, which was estimated based on the 85% percentile density score [22]. To analyze CT images, ITK-SNAP (www.itksnap.org), a software application used to segment structures in 3D medical images for setting a region of interest (ROI) [23]. Usually, 20 slices of the severe fibrosis were selected for calculating the 85% percentile density score, based on all voxel intensities in the ROI.

#### 10. Lung Myeloperoxidase (MPO) activity

To measure MPO activity, the tissue of the right lung was homogenized in 50 mmol/L PBS containing 0.5% hexadecylammonium bromide and 5 mmol/L EDTA (pH = 6.0). Then, the lung extracts were centrifuged at 12500 ×g for 20 min at 4°C to collect the supernatants, which were incubated with 50 mmol/L PBS containing 30% H_2_O_2_ and o-dianisidine dihydrochloride (167 mg/ml, Sigma–Aldrich, St.Louis, MO, United States). The change in absorbance at 460 nm over 3 min was recorded for the determination of MPO activity [24].

#### 11. HE, MT, and Sirius red staining

The fixed lung tissues (in 4% paraformaldehyde for 24 h) were dehydrated in an ethanol gradient and embedded in paraffin, followed by cutting the section at 5-μm thickness for HE, MT and Sirius red staining. The severity of PF was assessed by the percentage of pulmonary collagen-positive area (blue) in MT staining, and the percentage of pulmonary collagen-positive area (red) in Sirius red staining as well as the Ashcroft scale [25]. Image J 6.0 software (National Institutes of Health, Bethesda, MD, United States) was used for image data analysis.

#### 12. Immunohistochemical (IHC) analysis

The fixed lung tissues as mentioned above were embedded in paraffin, then sectioned at 5-μm thickness, and finally stained with the primary antibodies as follows: α-SMA (1:100, A17910, ABclonal), Collagen I (1:500, ab270993, Abcam) and Collagen III (1:500, 22734-1-AP, Proteintech) overnight at 4°C. On the next day, the slides were incubated with appropriate secondary antibodies at 37°C for 1 hour. IHC results were visualized using diaminobenzidine (DAB). At least 5 random microscopic fields were selected. Image J 6.0 software was used to segment the ROI and to calculate the intensity of IHC staining.

#### 13. Western blot analysis

The frozen lung tissues were homogenized in ice-cold radioimmunoprecipitation (RIPA) lysis buffer (Beyotime Institute of Biotechnology, China) to collect the lysate of the lung tissue after centrifugation (12000×g, 10 min at 4°C). The protein concentration of the lysate was determined using a Bicinchoninic Acid Protein Assay Kit (Beyotime Institute of Biotechnology, China). Equal amounts of protein (40 μg) were loaded and separated by 7.5–12% SDS-PAGE and then electrophoretically transferred onto polyvinylidene difluoride (PVDF) membranes (Millipore, Bedford, MA, United States). The membranes were blocked with 5% non-fat dry milk in Tris-buffered saline with 0.1% Tween-20 (TBS-T) for 1 hour at room temperature, followed by incubation with the primary antibodies as follows overnight at 4°C: anti-TGF-β1 (1:1,000 dilution, ab215715, Abcam), anti-Smad2 (1:2,000 dilution, ab40855, Abcam), anti-phospho-Smad2 (Ser465/Ser467) (1:1,000 dilution, ab280888, Abcam), anti-Smad3 (1:1,000 dilution, ab40854, Abcam), anti-phospho-Smad3 (Ser423/425) (1:500 dilution, AP0727, ABclonal), anti-PI3K (1:1,000 dilution, ab133595, Abcam), anti-phospho-PI3K (1:500 dilution, ab182651, Abcam), anti-Akt (1:3,000 dilution, #9272, CST), anti-phospho-Akt (1:1,000 dilution, #9272, CST), anti-NF-κB (1:3,000 dilution, #8242, CST), anti-phospho-NF-κB (1:500 dilution, #3033, CST), anti-TIMP-1 (1:1,000 dilution, 16644-1-AP, Proteintech), anti-MMP-9 (1:1,000 dilution, A0289, ABclonal), anti-fibronectin (1:1,000 dilution, ab268020, Abcam), anti-E-cadherin (1:1,000 dilution, A20798, ABclonal), anti-vimentin (1:1,000 dilution, A19607, ABclonal), α-SMA (1:500, A17910, ABclonal), anti-IL-6 (1:1,000 dilution, A0286, ABclonal), anti-TNF-α (1:1,000 dilution, A11534, ABclonal), and anti-IL-1β (1:1,000 dilution,A1112, ABclonal). On the next day, After washing with TBS-T thrice, the membranes were incubated with horseradish peroxidase-labeled goat anti-rabbit IgG (1: 2000 dilution; Biosynthesis Biotechnology Co., Ltd., Beijing, China) or goat anti-rat IgG (1:2,000 dilution; Biosynthesis Biotechnology Co., Ltd., Beijing, China) for 1 h. The bands were visualized by enhanced chemiluminescence (ECL) kit (GE Healthcare, United States) Image J 6.0 software was used for the analysis of western blotting data.

#### 14. Statistical analysis

In this study, GraphPad software (version 7.0) was used for all statistical analyses. All results were presented as mean ± SD. Multiple sample data were analyzed using one-way analysis of variance (ANOVA), followed by Dunnett’s t post-hoc test or Tukey’s test. If data were not normally distributed, raw data were analyzed by Kruskall-Wallis test, followed by Dunn’s multiple comparison test. *P* <0.05 was considered statistically significant.

## Results

### Part I. Network pharmacology-based analysis

#### 1. Identification of the bioactive components of QFHXD

**Fig 1:**
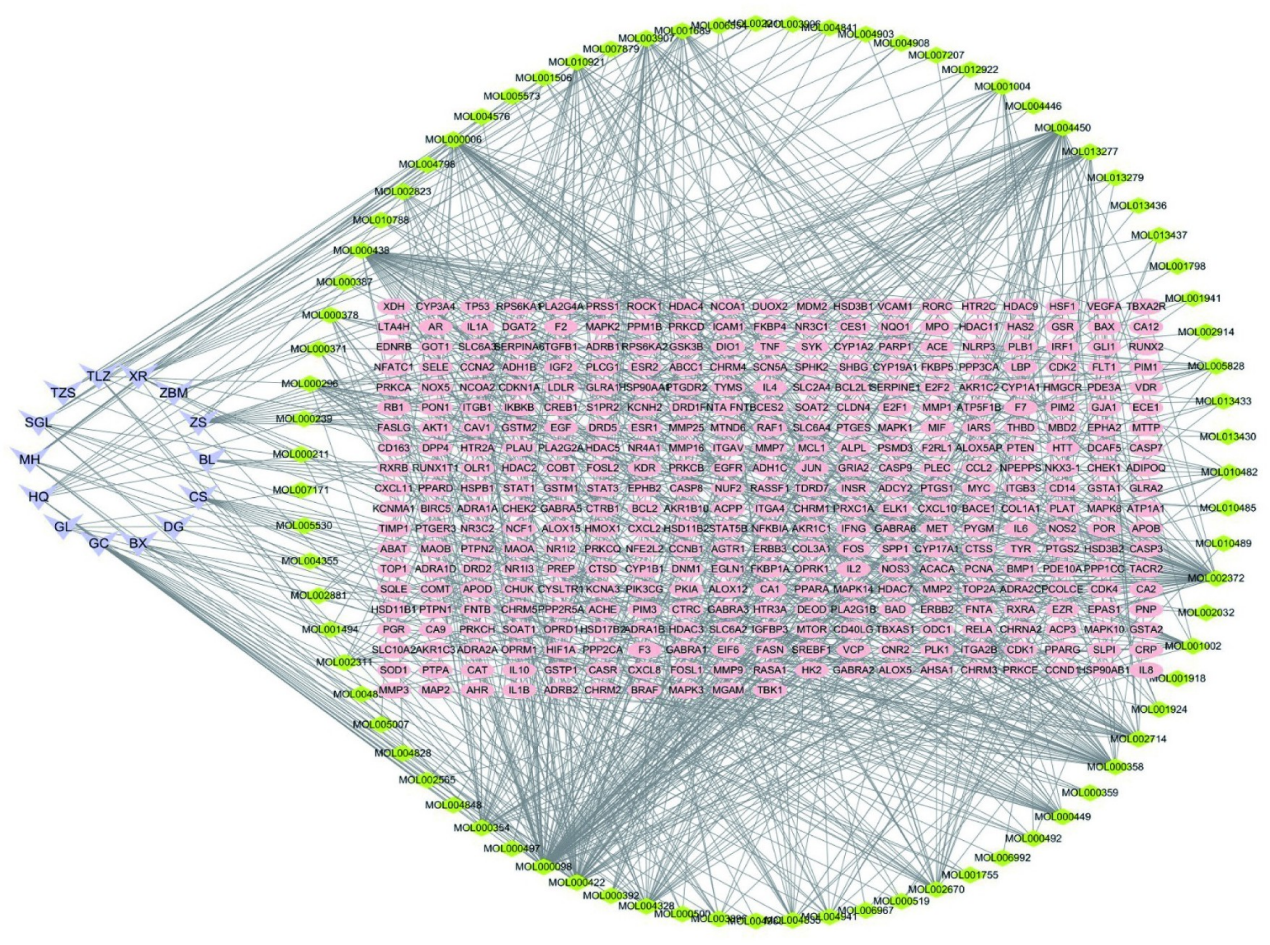
The “herb-component-target” interaction network related to the effects of QFHXD on PF, based on the network pharmacology analysis. In the network, the purple arrow represents the main herbs of QFHXD. The green and pink circles represent active components and targeted genes of QFHXD against PF, separately. HQ: *Astragalus membranaceus*, DG: *Angelica sinensis*, MH: *Ephedra sinica Stapf*, XR: *Prunus armeniaca*, BX: *Pinellia ternata*, GL: *Trichosanthes kirilowii Maxim*, TZS: *Pseudostellaria heterophylla*, TLZ: *Lepidium apetalum Willd*, CS: *Paeonia lactiflora Pall*, BL: *Areca catechu L*, ZS: *Citrus aurantium L*, ZBM: *Fritillaria thunbergii Miq*, SGL: *Luffa cylindrica Roem*, *and* GC: *Radix Glycyrrhizae*.

Total 79 active compounds in the constituents of QFHXD were identified, based on the criteria (OB≥30% and DL≥0.18), all of which were listed in S1 Table. Among those active compounds, some were considered as the vital active compounds, such as luteolin, quercetin, isorhamnetin, beta-sitosterol, sitosterol, and kaempferol. As shown in Fig 1, the“herb-compound-target”interaction network related to the effects of QFHXD on PF included 464 nodes and 991 edges. In this network, there were 14 herbs that can regulate multiple compounds, and are associated with considerable targets. For instance, the estrogen receptor could be modulated by a range of compounds, such as ellagic acid, coniferin, and leucopelargonidin, suggesting that the active compounds derived from QFHXD might effectively treat pulmonary fibrosis through multiple targets. The relationships between herbs, active compounds and targets as well as the potential pharmacological effects of QFHXD on PF were visually illustrated in this network (Fig 1).

#### 2. Identification of the targets related to the effects of QFHXD on PF

**Fig 2:**
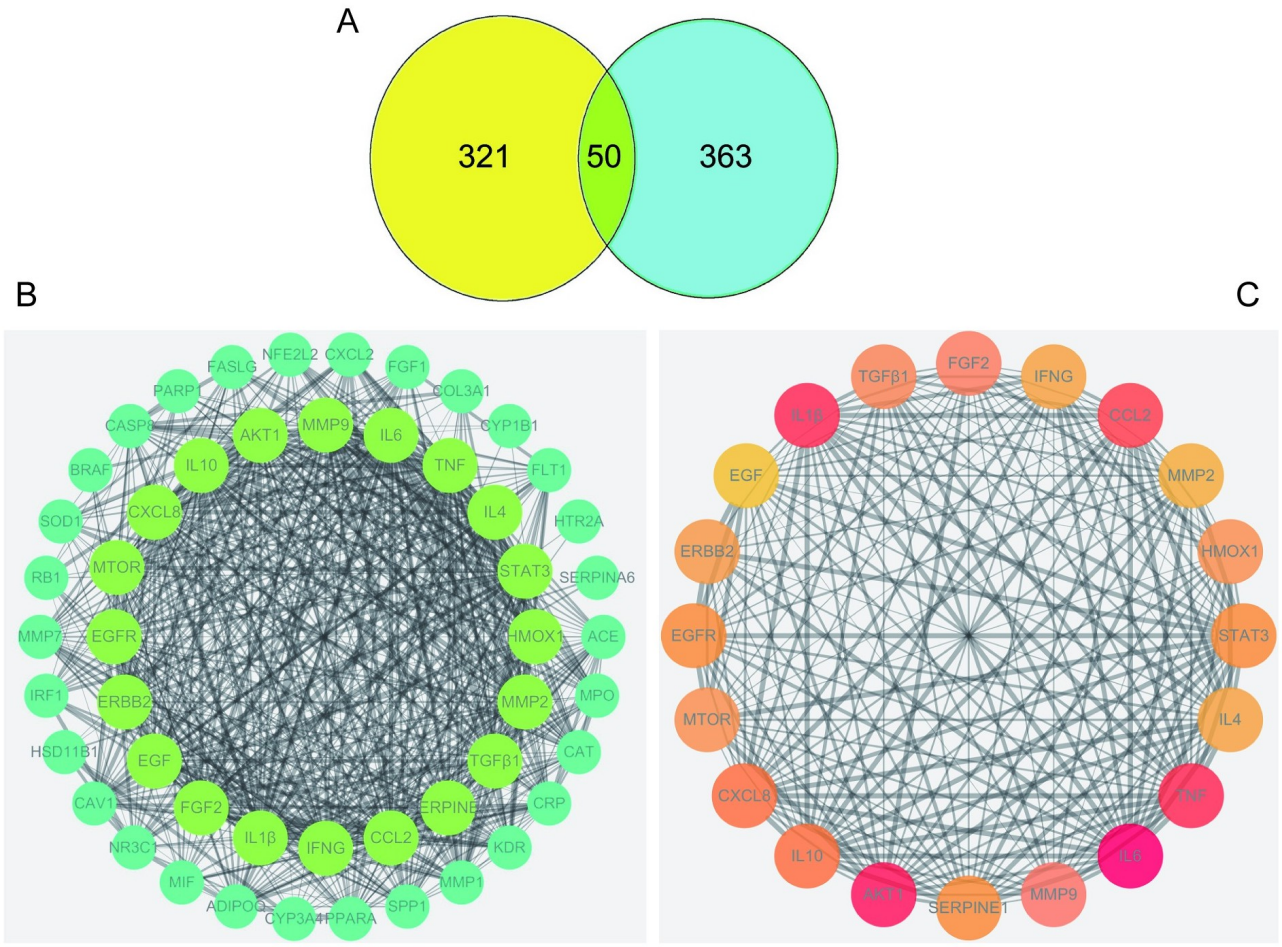
Venn diagram and PPI networks of the potential protein targets of the treatment with QFHXD. (A) Venn diagram of the potential anti-PF targets. (B) PPI network of the protein targets. The circles represent the overlapped targets related to the effects of QFHXD on PF. (C) The top 20 core nodes in the PPI network are presented. The color depth and size of those core nodes are dependent on their degree values.

Among the 79 candidate bioactive components, 1559 protein targets were retrieved from TCMSP database and Swiss TargetPrediction. After eliminating the overlapping and insignificant targets, 371 protein targets were obtained for further analyses. 413 PF-related human genes were collected from Disgenet, Drugbank, OMIM and TTD databases. As a result, 50 target genes were identified affected by pulmonary fibrosis and regulated by QFHXD, indicating the close relationship between the fourteen herbs of QFHXD and pulmonary fibrosis disease (Fig 2A).

#### 3. PPI network related to the effects of QFHXD on PF

To find the most highly connected nodes, and the overlapped protein targets, the analysis of PPI network was also performed. As illustrated in Fig 2B, the PPI network related to the effects of QFHXD on PF covered 50 nodes and 634 edges, suggesting that QFHXD exerts its explicit therapeutic effects on pulmonary fibrosis through multiple protein targets. In addition, this network was constructed, based on the degrees, betweenness and closeness of the core nodes of QFHXD (Fig 2C). The top 20 core nodes were shown in Table 1, among which, IL-6, AKT, TNF, IL-1β, and IL-10 were at the top position, refer to the major targets involved in the treatment of pulmonary fibrosis.

**Table 1:** The top 20 core target PPI network of QFHXD in treating PF.

Gene name	Target	Degree
IL6	Interleukin-6	45
AKT1	RAC-alpha serine/threonine-protein kinase	43
TNF	Tumor necrosis factor	42
IL1β	Interleukin-1 beta	42
CCL2	C-C motif chemokine 2	41
IL10	Interleukin-10	39
CXCL8	Interleukin-8	39
STAT3	Signal transducer and activator of transcription 3	38
MMP9	Matrix metalloproteinase-9	38
EGFR	Epidermal growth factor receptor	38
EGF	Pro-epidermal growth factor	36
MMP2	72 kDa type IV collagenase	34
IL4	Interleukin-4	33
IFNG	Interferon gamma	33
ERBB2	Receptor tyrosine-protein kinase erbB-2	32
SERPINE1	Plasminogen activator inhibitor 1	32
TGFβ1	Transforming growth factor beta-1	32
MTOR	Serine/threonine-protein kinase mTOR	31
FGF2	Fibroblast growth factor 2	31
HMOX1	Heme oxygenase 1	31

#### 4. GO and KEGG pathway enrichment analysis

**Fig 3:**
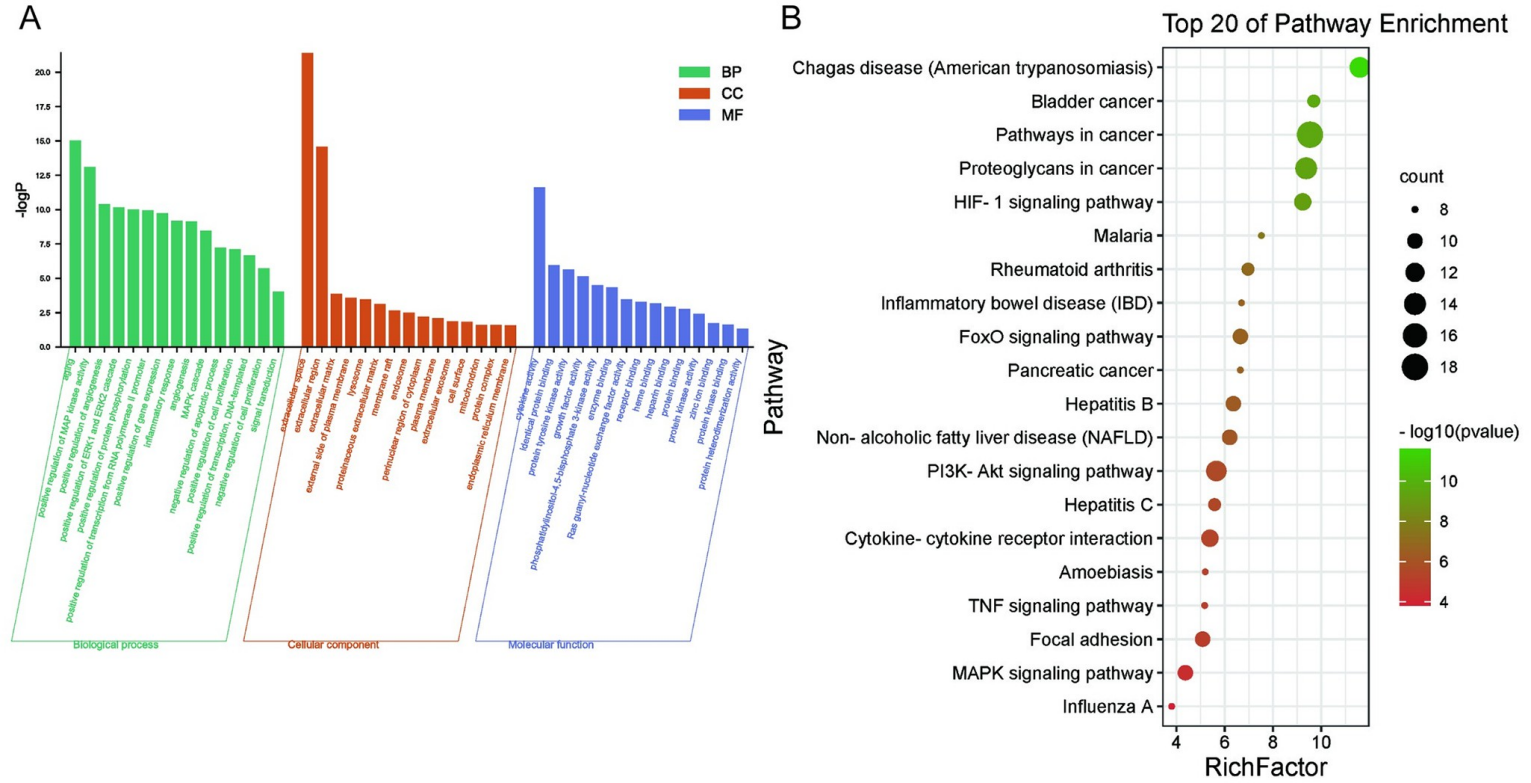
Enrichment analysis of the potential targets related to the effects of QFHXD on PF. (A) The top 15 biological process (BP), cellular component (CC), and molecular function (MF) terms. (B) The top 20 KEGG pathways.

To investigate the biological characteristics of the putative targets related to the effects of QFHXD on PF, the GO and pathway enrichment analysis for the involved targets were performed, using the functional annotation tool of DAVID Bioinformatics Resources 6.8. Results revealed total 331 biological processes (BP), 26 cellular components (CC), and 46 molecular function (MF) terms, all of which could be identified, because they met the requirements of Count≥ 2 and P value≤ 0.05. The top 15 significantly enriched GO terms in the categories of BP, CC, and MF were shown in Fig 3A, suggesting that QFHXD may inhibit the progression of pulmonary fibrosis mainly through up-regulation of the aging process, MAP kinase activity, angiogenesis, ERK1 and ERK2 cascade, and protein phosphorylation. To explore the mechanisms underlying the effects of QFHXD on PF, the KEGG pathway enrichment analysis was conducted. The top 20 significantly enriched pathways related to the effects of QFHXD on PF were found (Fig 3B), according to the enrichment target counts and p values. As is shown in Table 2, the therapeutic effects of QFHXD on PF were involved in the regulation of the signaling pathways as follows: PI3K/Akt, HIF-1, FoxO, MAPK, and TNF.

**Table 2:** The top 20 KEGG pathway.

Pathway ID	Pathway name	P value	Count
hsa05200	Pathways in cancer	2.94E-10	18
hsa05205	Proteoglycans in cancer	4.24E-10	14
hsa05142	Chagas disease (American trypanosomiasis)	2.52E-12	13
hsa04151	PI3K-Akt signaling pathway	2.23E-06	13
hsa04066	HIF-1 signaling pathway	5.89E-10	11
hsa04060	Cytokine-cytokine receptor interaction	4.12E-06	11
hsa04068	FoxO signaling pathway	2.25E-07	10
hsa05161	Hepatitis B	4.41E-07	10
hsa04932	Non-alcoholic fatty liver disease (NAFLD)	6.23E-07	10
hsa04510	Focal adhesion	8.25E-06	10
hsa04010	MAPK signaling pathway	4.27E-05	10
hsa05219	Bladder cancer	2.05E-10	9
hsa05323	Rheumatoid arthritis	1.09E-07	9
hsa05160	Hepatitis C	2.64E-06	9
hsa05144	Malaria	3.02E-08	8
hsa05321	Inflammatory bowel disease (IBD)	2.02E-07	8
hsa05212	Pancreatic cancer	2.26E-07	8
hsa05146	Amoebiasis	6.40E-06	8
hsa04668	TNF signaling pathway	6.82E-06	8
hsa05164	Influenza A	1.57E-04	8

#### 5. Analysis of the target-pathway network

**Fig 4:**
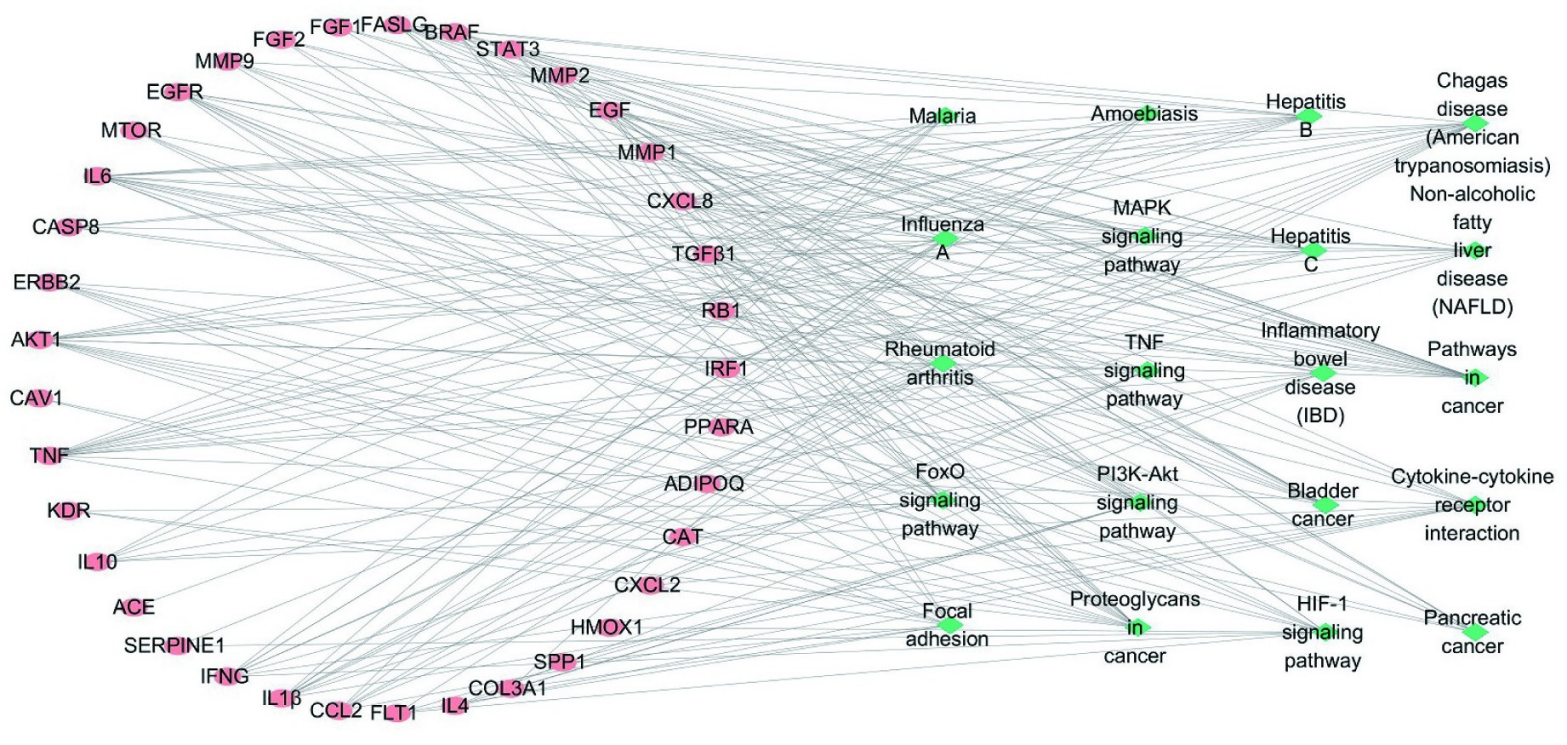
The “target-pathway” network. The rose-red circle represents the potential targets related to QFHXD treatment. The green rhombus represents the main targeted pathways. The gray edges represent the interaction between the potential targets and their enriched pathways.

To illustrate the relationship between the targets related to the effects of QFHXD on PF and the involved signaling pathways, the KEGG target-pathway network was established, based on the 20 top KEGG pathways. In this network, there were 57 nodes (37 targets and 20 pathways) and 204 edges (Fig 4). Among these potential pathways, the most significant one with the highest enrichment target count was the cancer-related pathway. Based on the data from comprehensive analysis using the integrated drug target prediction; GO and KEGG pathway enrichment; and the target-pathway network, we speculated that IL-6, AKT1, TNF, IL-1β, STAT3, MMP-9, and TGF-β1 might be the key targets, which play critical roles in the inhibitory effects of QFHXD on the progression and exacerbation of PF.

### Part II. Experimental validation

#### 6. QFHXD alleviated BLM-induced pulmonary fibrosis in rats

**Fig 5:**
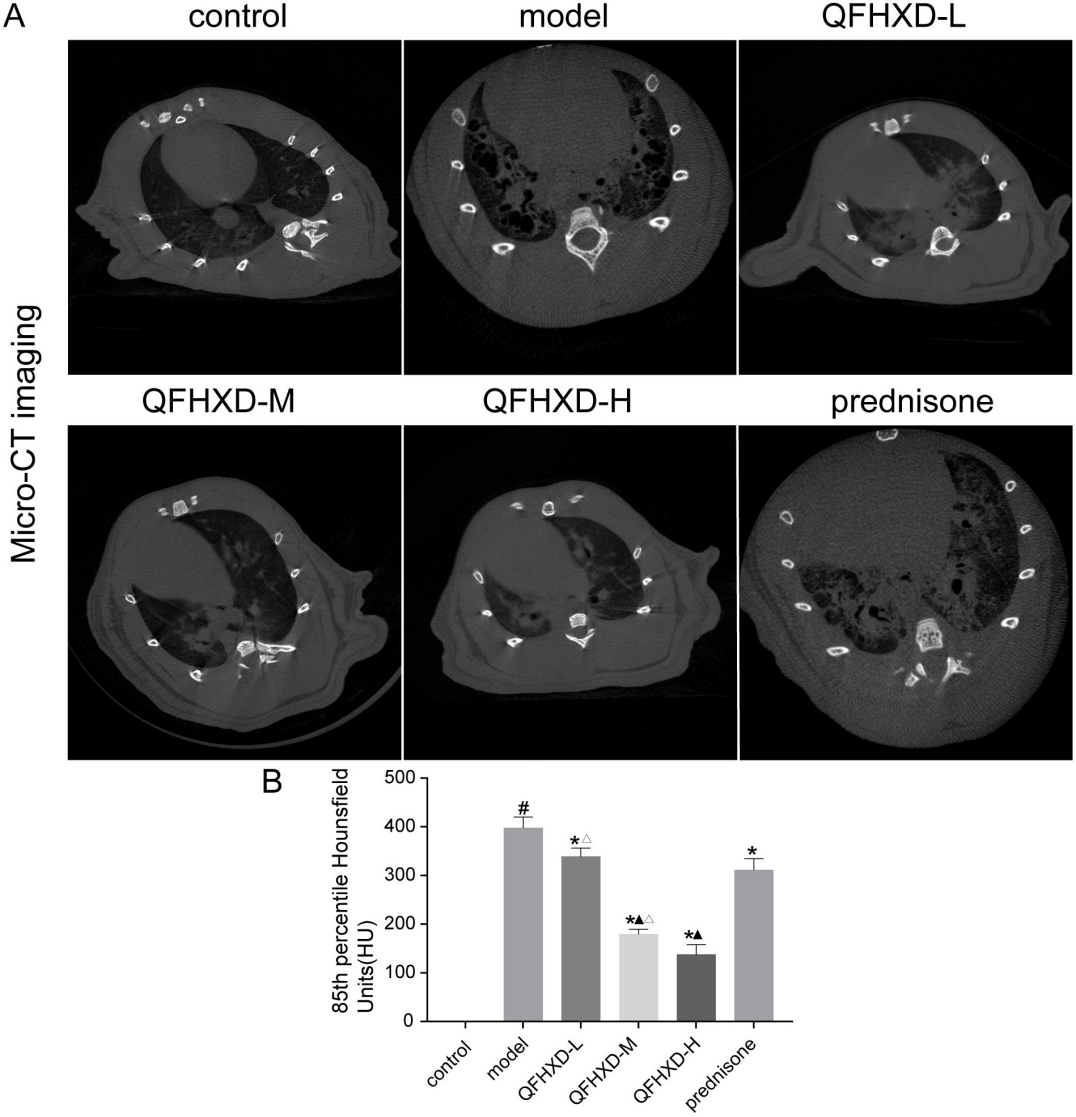
The representative micro-CT images and fibrotic score in each group. (A) The representative micro-CT images in each group. (B) The quantity of micro-CT in rats by lung density parameter of Perc85. The data are presented as mean ± SD (n = 3 per group). ^#^p<0.05 versus control group,*p<0.05 versus model group, ^▲^p<0.05 versus prednisone group, ^Δ^p<0.05 versus QFHXD-H group.

**Fig 6:**
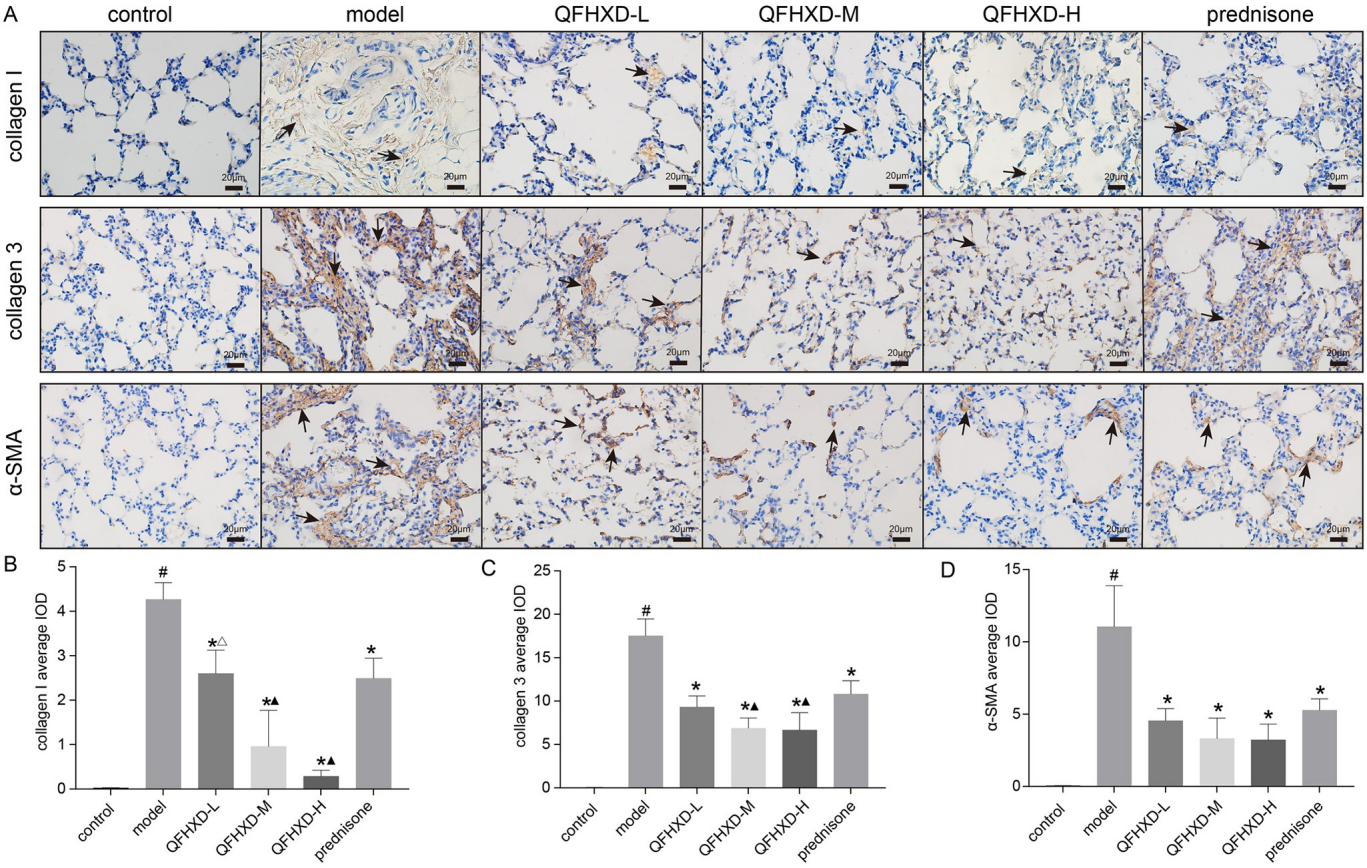
QFHXD and prednisone reduced the expression of collagen 1, collagen 3 and α-SMA in BLM-induced PF rats. (A) Immunohistochemical staining of lung sections in each group. Magnification, ×400. Scale bar, 20μm. (B) Average IOD of collagen1 in each group. (C) Average IOD of collagen 3 in each group. (D) Average IOD of α-SMA in each group. The data are presented as mean ± SD (n = 3 per group). IOD, integral light density. ^#^p<0.05 versus control group, *p<0.05 versus model group, ^▲^p<0.05 versus prednisone group, ^Δ^p<0.05 versus QFHXD-H group.

To evaluate the effect of QFHXD on BLM-induced pulmonary fibrosis in rats, the lung of the anesthetized rats were scanned by a micro-CT. system, on the 28^th^ day after the QFHXD and prednisone treatment. The CT images were analyzed by a semi-quantitative method based on a four-point ranking scale [26]. The 85% percentile density score was used to determine the degree of fibrosis [27]. Immunohistochemical (IHC) analysis was also used to evaluate the changes in collagen fibers in lung tissues. As shown in Fig 5, the intratracheal administration of BLM can induce a remarkable increase in collagen deposition, as compared to the lungs in control group. QFHXD treatment can significantly reduce the α-SMA, collagen I and collagen III at high, middle, and low doses, as same as prednisone treatment (Fig 6A–6C). In particular, QFHXD treatment showed better improvents in reducing the collagen I and collagen III than prednisone treatment. Taken together, these results implied that QFHXD and prednisone treatment can prevent bleomycin-induced pulmonary fibrosis.

#### 7. QFHXD attenuated BLM-induced pathological changes in the lung of rats

**Fig 7:**
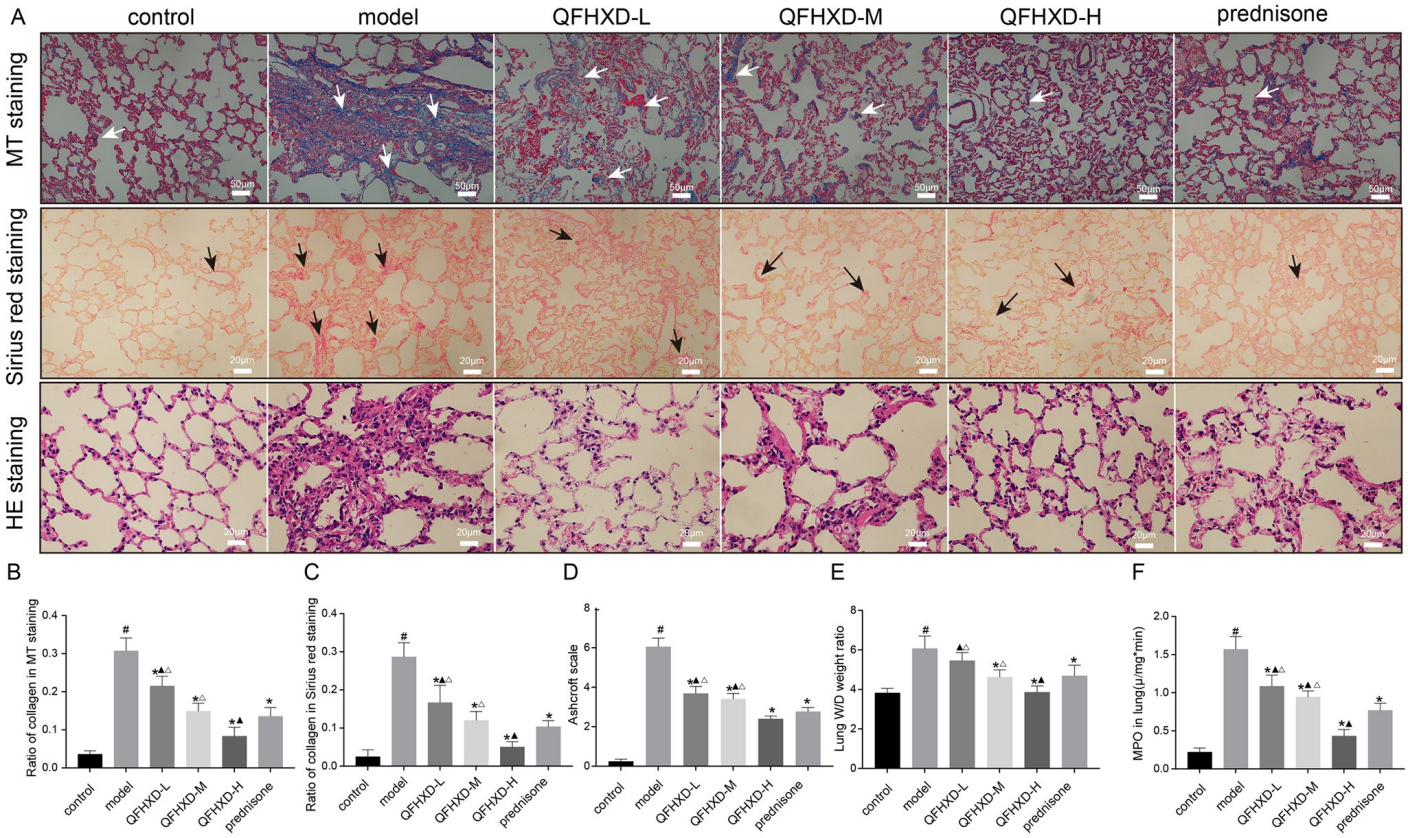
QFHXD and prednisone alleviated pathological changes in BLM-induced PF rats. (A) Masson’s trichrome staining,Sirius red staining, and H&E staining of lung sections in each group. Magnification were ×200, ×400,and ×400, respectively. Scale bar were 50μm, 20μm, and 20μm, respectively. (B) Ratio of collagen in MT staining in each group. (C) Ratio of collagen in Sirius red staining in each group. (D) Ashcroft scale in each group. (E) Lung W/D weight ratio in each group. (F) MPO in lung in each group. The data are presented as mean ± SD (n = 3 per group). MT, Masson’s trichrome. ^#^p<0.05 versus control group, *p<0.05 versus model group, ^▲^p<0.05 versus prednisone group, ^Δ^p<0.05 versus QFHXD-H group.

The histopathological changes in the lung tissues of the experimental rats were observed and evaluated by HE staining. The results of HE staining showed that there were significant histopathological changes in lung tissues collected from the model group (BLM only) (Fig 7), such as the distinct increases in minimal fibrous thickening of alveolar or bronchiolar vessels, alveolar destruction, and inflammatory cells infiltration. Meanwhile, the treatment of QFHXD at all three doses or prednisone treatment can induce a decrease in the infiltration of the inflammatory cells, and interstitial thickness. Similarly, MT and Sirius red staining revealed that the treatment with either QFHXD or prednisone can alleviate the collagen deposition (Fig 7A–7C). Ashcroft scale results also indicated that the fibrosis severity in rats was reduced by the treatment with either QFHXD or prednisone (Fig 7D). As shown in Fig 7E, the intratracheal administration of BLM resulted in a significant increase in lung W/D weight ratio in the experimental rats. Whereas, the BLM-induced increased W/D weight ratio was significantly reversed by treatment with QFHXD (medium and high-dose) or prednisone. The measurement of MPO activity also indicated that BLM treatment can induce an increase in MPO levels in the lung tissue extracts, but the treatment with QFHXD at all three doses or prednisone can remarkably attenuate the BLM-induced elevated MPO levels (Fig 7F). Additionally, QFHXD treatment at all three doses had stronger protective effects than that in the prednisone treatment group. Besides, the higher efficiency of the treatment of QFHXD at high-dose was observed, as compared to that in its low-dose and medium dose treatment.

#### 8. QFHXD inhibited the BLM-mediated EMT

**Fig 8:**
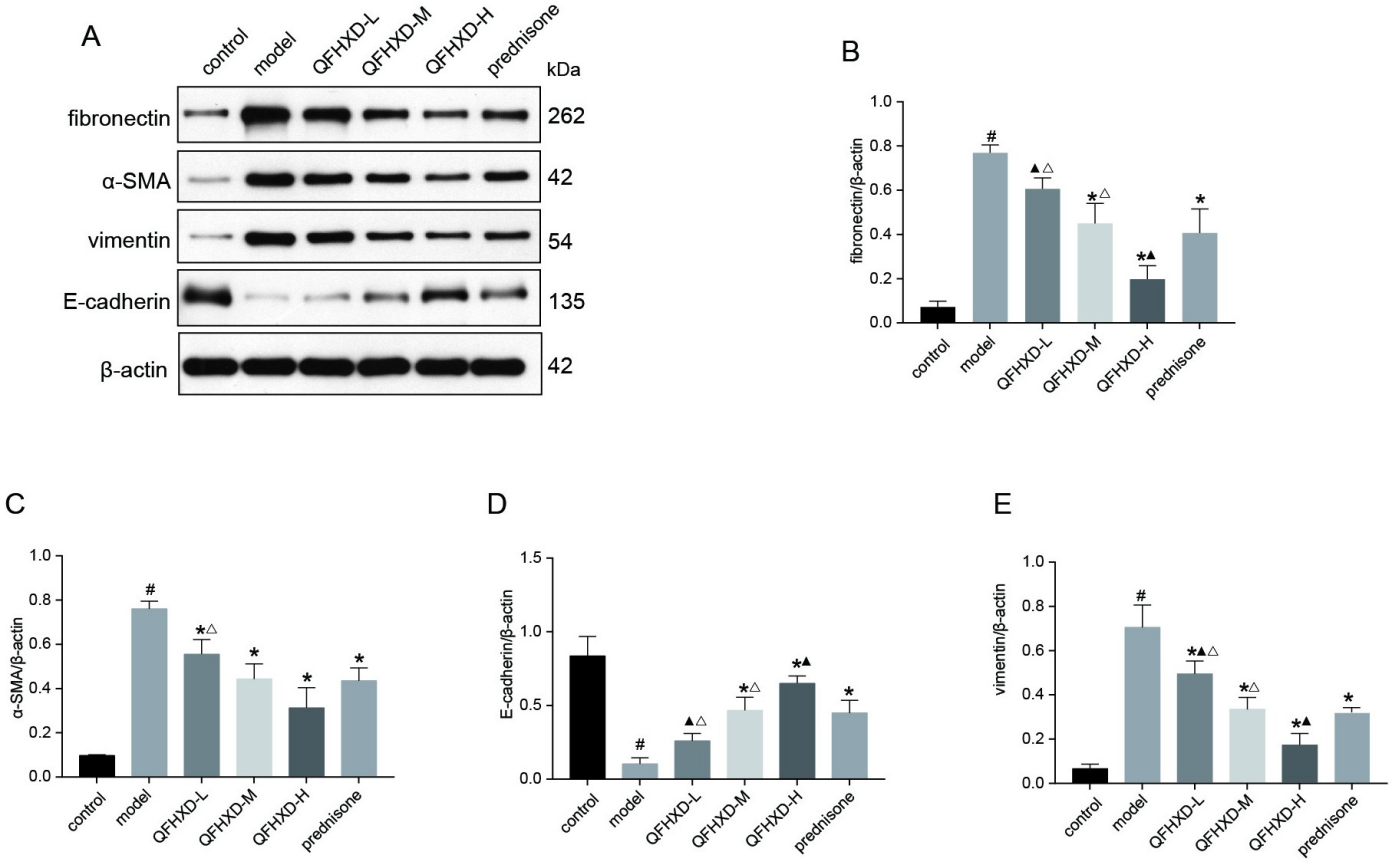
Effects of QFHXD and prednisone treatment on EMT in BLM-induced PF rats. (A) α-SMA, fibronectin, E-cadherin, and vimentin protein expression in lung tissues in each group as measured by western-blot analysis. (B, C, D, E) The densitometry values for the proteins were normalized to those of β-actin. All data represent the mean ± SD of three independent experiments performed in triplicate. ^#^p<0.05 versus control group, *p<0.05 versus model group, ^▲^p<0.05 versus prednisone group, ^Δ^p<0.05 versus QFHXD-H group.

EMT, a process where epithelial cells lose epithelial proteins, such as E-Cadherin, is closely involved in the pathogenesis of pulmonary fibrosis [28]. To investigate whether EMT occurred in the animal model of BLM-induced PF, the expression levels of several classic EMT markers in the lung tissues, including α-SMA, vimentin, fibronectin, and E-cadherin were examined by western-blot analysis. As shown in Fig 8, the BLM treatment can cause a significant decrease in the expression level of E-cadherin, compared with that in the control group, whereas the expression levels of α-SMA, vimentin, and fibronectin were significantly increased by BLM treatment. Treatment with QFHXD and prednisone can reverse the BLM-mediated changes in the expression levels of these proteins, in particular, the high-dose QFHXD treatment had strongest anti-EMT effects.

#### 9. QFHXD rescued MMP-9/TIMP1 imbalance and prevented inflammation via inactivation of PI3K/Akt/ NF-κB pathway

**Fig 9:**
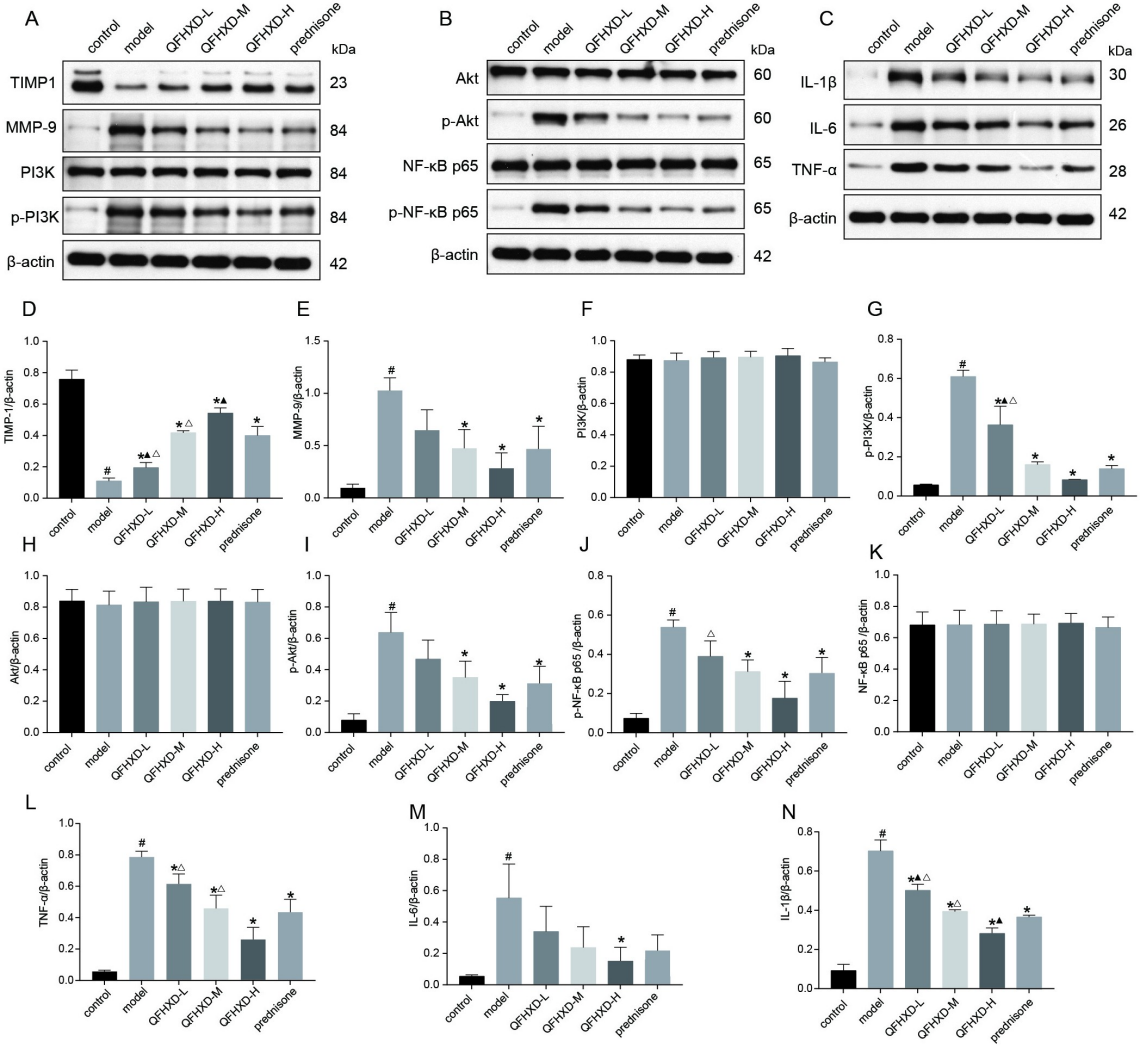
Effects of QFHXD and prednisone treatment on MMP-9/TIMP1 balance and inflammation in BLM-induced PF rats. (A, B, C) MMP-9, TIMP1, PI3K, p-PI3K, Akt, p-Akt, NF-κB p65, p-NF-κB p65, TNF-α, IL-6, and IL-1β protein expression in lung tissues in each group as measured by western-blot analysis. (D-N) The densitometry values for the proteins were normalized to those of β-actin. All data represent the mean ± SD of three independent experiments performed in triplicate. ^#^p<0.05 versus control group, *p<0.05 versus model group, ^▲^p<0.05 versus prednisone group, ^Δ^p<0.05 versus QFHXD-H group.

MMP-9 overexpression can degrade all components of the extracellular matrix and nonmatrix proteins, thereby prompting pulmonary fibrosis [29]. Moreover, the tissue inhibitors of metalloproteinases (TIMPs) play a vital role in the inhibition of tissue organization and fibrosis [30]. To explore the mechanisms underlying anti-EMT effects of QFHXD, we investigated whether QFHXD and prednisone treatment can rescue the MMP-9/TIMP1 imbalance in the animal model of BLM-induced PF. As presented in Fig 9A, 9D and 9E, the expression level of MMP-9 protein was significantly elevated in the model group, meanwhile, TIMP1 expression was remarkably downregulated. Treatment with QFHXD and prednisone can significantly reverse this imbalance caused by BLM. In particular, the high-dose QFHXD treatment had strongest effect on this imbalance. Since the PI3K/Akt/NF-κB pathway was reported involved in the inflammation and fibrosis in PF [31], the effect of QFHXD treatment on this signaling pathway was also evaluated in our study, including detection of inflammatory cytokines, such as TNF-α, IL-6, and IL-1β, as well as the PI3K/Akt/NF-kB pathway-related molecules. As shown in Fig 9A–9C and 9F–9N), the levels of p-PI3K, p-Akt, p-NF-κB p65, TNF-α, IL-6, and IL-1β were significantly elevated in the model group of the animal model. Whereas, treatment with QFHXD at all doses and prednisone can remarkably decrease the levels of p-PI3K, p-Akt, p-NF-κB p65 and inflammatory cytokines, which were elevated by BLM.

#### 10. QFHXD ameliorated the BLM-induced pulmonary fibrosis via probably inhibiting TGF-β1/Smad2/3 signaling pathway

**Fig 10:**
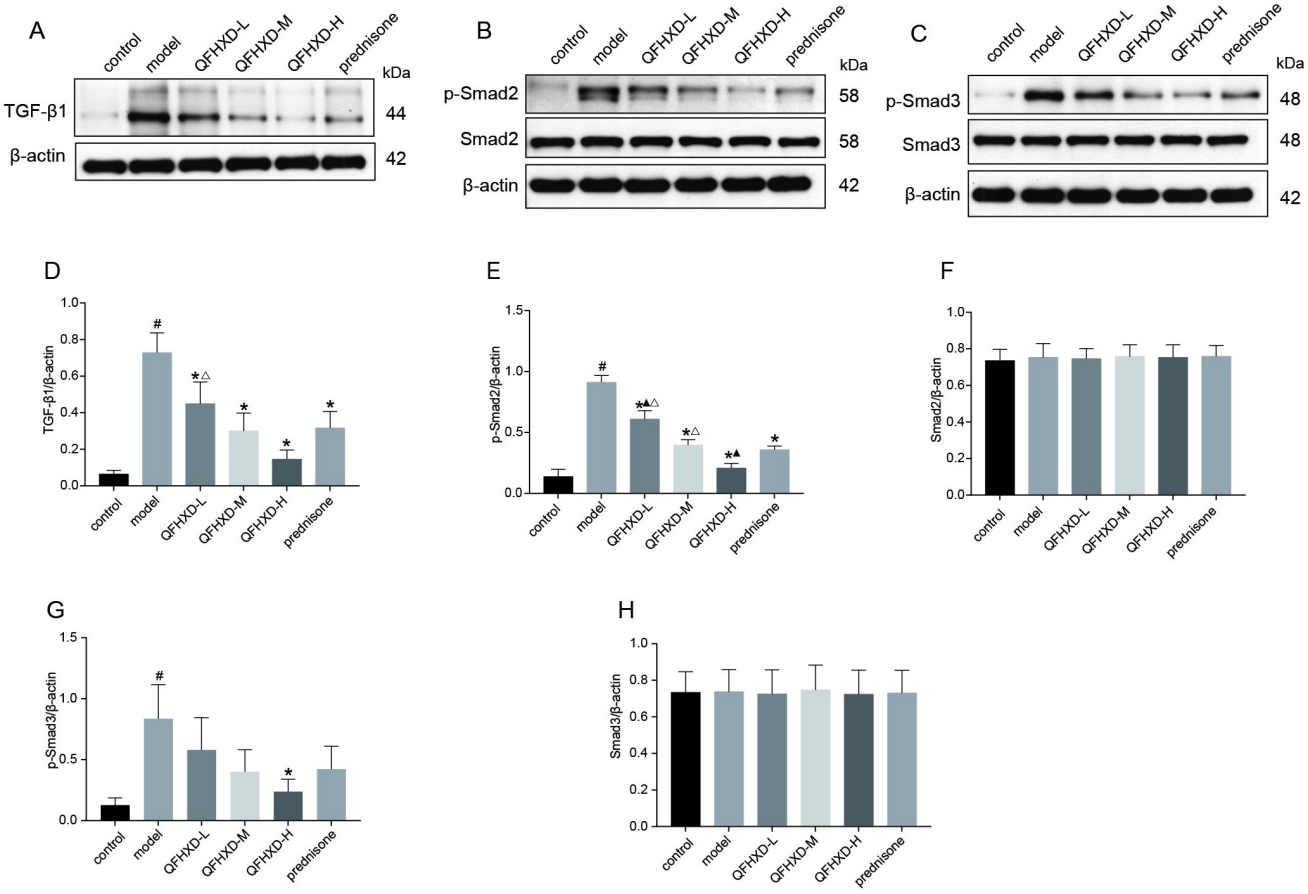
Effects of QFHXD and prednisone treatment on TGF-β1/Smad2/3 signaling in BLM-induced PF rats. (A, B, C) TGF-β1, p-Smad2, Smad2, p-Smad3, and Smad3 protein expression in lung tissues in each group as measured by western-blot analysis. (D-H)The densitometry values for the proteins were normalized to those of β-actin. All data represent the mean ± SD of three independent experiments performed in triplicate. ^#^p<0.05 versus control group, *p<0.05 versus model group, ^▲^p<0.05 versus prednisone group, ^Δ^p<0.05 versus QFHXD-H group.

Since the TGF-β1/Smad pathway acts a vital role in EMT in pulmonary fibrosis [32], we also examined the activity of TGF-β1/smad pathway in the lungs of the BLM-treated rats. As shown in Fig 10, BLM treatment can significantly increase the levels of TGF-β1 protein, and phosphorylated Smad2 and Smad3, implying an activation of TGF-β1/Smad2/3 signaling pathway caused by BLM. On the other hand, treatment with QFHXD and prednisone significantly inhibited the BLM-induced activation of TGF-β1/Smad2/3 pathway in a dose-dependent manner. Our findings suggested that QFHXD can protect rats against the BLM-induced PF, partly through suppression of TGF-β1/Smad2/3 signaling pathway.

## Discussion

Our study identified the bioactive compounds in the constituents of QFHXD and explored the QFHXD anti-PF molecular mechanisms using network pharmacology analysis. The predicted mechanisms were further evaluated in the animal model of BLM-induced PF. Based on GO analysis and KEGG pathway analysis, we predicted that PI3K/Akt signaling pathway and TGF-β1/Smad2/3 pathway probably played crucial roles in QFXHD-induced protective effects in PF. In the present study, the rats with bleomycin-induced PF were treated with QFHXD at low, medium and high doses or prednisone to validate the protective role of QFHXD. The fibrosis severity of lung tissues was assessed using several methods, including in-vivo Micro-CT, HE staining, Masson staining and Sirius red staining. Results showed that the intratracheal administration with bleomycin caused remarkable increase in collagen deposition and inflammatory cytokines in rats. QFHXD significantly reduced collagen deposition, and inhibited the expressions of inflammatory proteins and EMT process at least partly via inhibiting the PI3K/Akt/ NF-κB pathway-involved inflammation and TGF-β1/Smad2/3 pathway. Our findings are consistent with the reports from other similar studies [33–37]. Our findings revealed a potential molecular mechanism underlying the QFHXD anti-PF effects.

MMP-9 can prompt the development of PF in multiple ways. TIMP-1, a tissue inhibitor of MMP-9 counteracts the effects of MMP-9 and is associated with ECM turnover and tissue remodeling [38]. An interruption in the balance between MMP-9/TIMP1 has been implicated in the development of PF [39]. In our study, bleomycin treatment up-regulated MMP-9 expression, but downregulated TIMP1 expression in the rat model, this was consistent with other’s findings [34, 40]. In addition, QFHXD treatment at all three doses could restore the dysregulated balance of MMP-9/TIMP1 caused by BLM.

It is widely accepted that EMT is closely correlated with the pathogenesis of many several lung diseases ranging from asthma, COPD, pulmonary fibrosis to lung cancer [41]. Multiple pathways have been demonstrated to trigger EMT, among which TGF-β1 signaling serves as an essential role in prompting collagen accumulation and the progression of lung fibrosis [42, 43]. It has been found that TGF-β1 expression is upregulated in lung tissues of the PF patients and rodent fibrotic lungs [44]. TGF-β1 can promote the EMT process of PF through regulation of signaling pathways, including the Smad, JNK, p38, Wnt/β and ERK signaling pathways [45]. A large body of experimental evidence supported that inactivation of TGF-β1/Smad signaling pathway could reduce EMT and block myofibroblast activation and collagen accumulation, leading to the protection of the tissues against PF [43, 46–48]. Our study revealed that BLM treatment could induce the activation of TGF-β1/Smad2/3 signaling pathway, by which EMT is developed. This result was consistent with other’s reports. An increase in the E-cadherin expression and a decrease in the expression of α-SMA, vimentin, and fibronectin were found in the QFHXD-treated rats, in which EMT was significantly inhibited.

Mounting evidence suggested that inflammatory reactions, including the overproduction of pro-inflammatory cytokines and increased infiltration of inflammatory cells, can induce PF [12]. Our study also found that the expressions of TNF-α, IL-6, IL-1β and MPO level were increased in BLM-treated rats, in accordance with other studies using the same animal model [34]. It is widely accepted that inflammation response contributes to the development of EMT, which plays a crucial role in driving the PF development [49, 50]. It has been reported that TNF-α induced EMT was through activation of Akt/NF-κB (nuclear factor kappa B) pathway in a renal allograft interstitial fibrosis [51]. In addition, IL-1β significantly upregulated the expressions of MMP-2 as well as EMT markers, such as vimentin, α-SMA, fibronectin [52]. These evidences indicated that targeting inflammation could be a potential approach for inhibiting the progression of EMT related to PF development. In the present study, we found that QFHXD treatment significantly decreased the levels of TNF-α, IL-6, IL-1β and MPO, and alleviated the degree of pulmonary fibrosis as evidenced by in vivo Micro-CT analysis, H&E staining, MT staining and Sirius red staining, which was at least partly via downregulating PI3K/Akt/NF-κB pathway. Mounting evidence revealed that PI3K/Akt signaling-mediated-inflammation may be responsible for various fibrosis diseases, including lung fibrosis [31, 53, 54], liver fibrosis [55], and intestinal fibrosis [56]. Therefore, inactivation of PI3K/Akt pathway attenuated PF progression via relieving inflammation and progression of EMT [57]. Consistently, the study on the anti-PF effects of Qingfei Paidu decoction (QFPD), a well-known Chinese herbal formula for the management of patients diagnosed with COVID-19, also indicated that QFPD can effectively alleviate the BLM-induced inflammation and collagen deposition in mice [58]. Both QFHXD and QFPD, two anti-PF herbal formulas share similar components including *Ephedra sinica Stapf*, *Prunus armeniaca*, *Citrus aurantium*, *Pinellia ternate*. Mass spectrometry and transcriptomic analysis further confirmed that the therapeutic effects of QFPD may be attributed to the anti-inflammatory effects. The inactivation of signaling pathways, such as Toll-like receptor 4, NF-κB, and MAPK might be associated with a decreased release of IL-8, IL-1β, and TNF-α, which contributed to anti-inflammatory effects of QFPD [59]. In the present study, we found that the anti-inflammatory effects of QFHXD was associated with inactivation of PI3K/Akt/NF-κB pathway. However, it still needs to further validate the bioactive components of QFHXD using bioinformatics analysis and multi-omics technologies, including transcriptomics, proteomics, and metabolomics, which may broaden the understanding of the anti-PF effects of QFHXD.

## Conclusion

**Fig 11:**
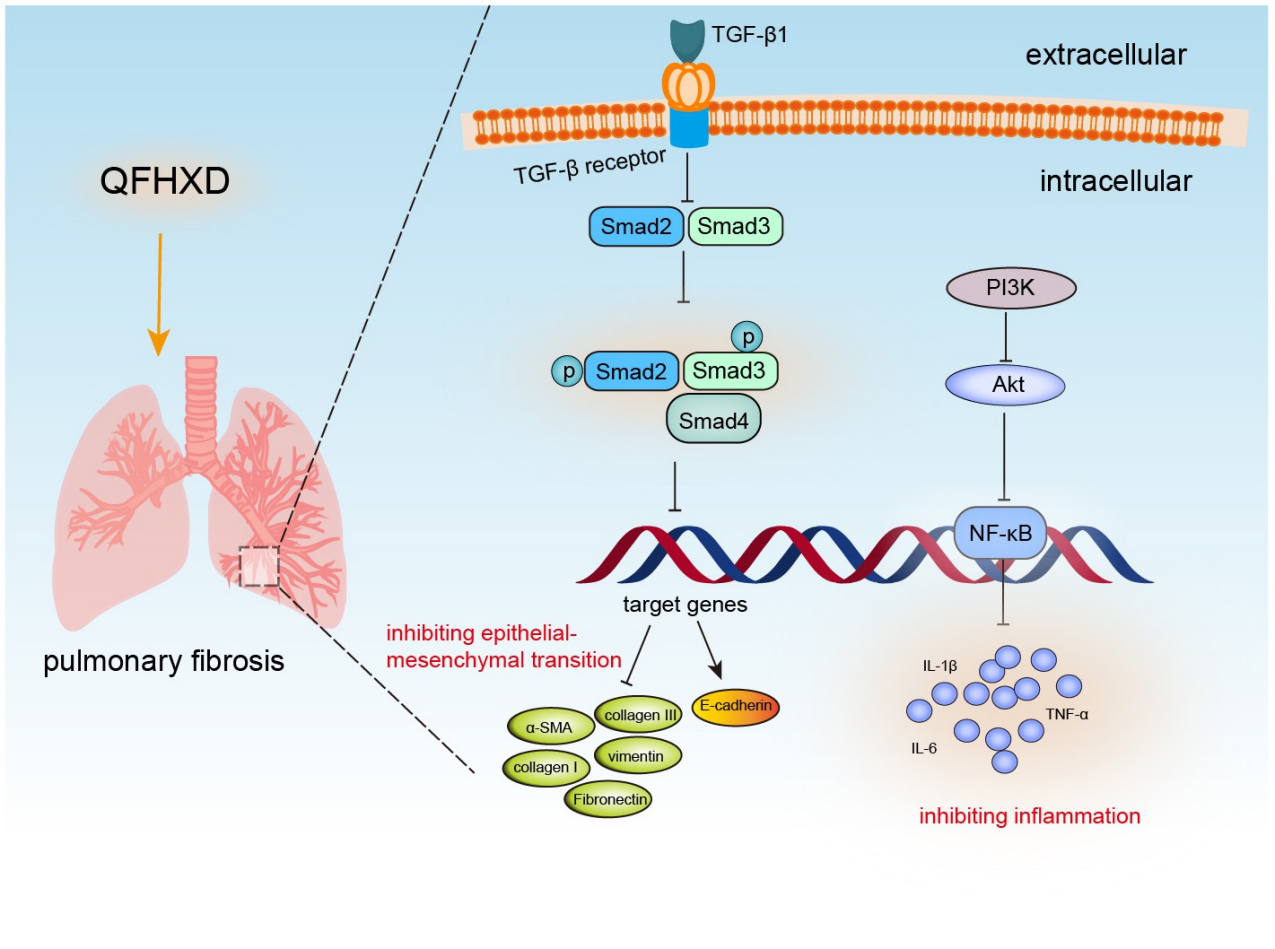
Schematic diagram showing how QFHXD alleviates BLM-induced pulmonary fibrosis by regulating PI3K/Akt/NF-κB and TGF-β1/Smad2/3 signaling pathways.

In summary, our study demonstrated the anti-PF effects of QFHXD in the rat model of BLM-induced PF, though the mechanism of the inhibition of EMT and inflammation, especially stronger protective effects of QFHXD at its high-dose. Moreover, inactivation of PI3K/Akt/NF-κB pathway and TGF-β1/Smad2/3 pathway may be the mechanisms underlying the anti-PF effects of QFXHD (Fig 11). Thus, QFHXD could be a promising therapeutic approach for treating pulmonary fibrosis.

## Supporting information

S1 Table Identified active compounds. (DOC)

S1 File Raw data for Figs 5–10. (XLSX)

S1 Raw image Original uncropped and unadjusted images for Figs 8–10. (PDF)

## Abbreviations

BP: biological processes
CC: cellular components
DAB: Diaminobenzidine
DL: Drug-likeness
ECL: enhanced chemiluminescence
ECM: Extracellular matrix
EMT: Epithelial-mesenchymal transition
GO: Gene ontology
IHC: Immunohistochemical
KEGG: Kyoto Encyclopedia of Genes and Genomes
MF: molecular function
MPO: Myeloperoxidase
OB: Oral bioavailability
PF: Pulmonary fibrosis
PPI: Protein-protein interactions
PVDF: polyvinylidene difluoride
QFHXD: Qing Fei Hua Xian Decotion
RIPA: Radioimmunoprecipitation
ROI: Region of interest
TCMSP: The traditional Chinese medicine systems pharmacology database
TIMPs: tissue inhibitors of metalloproteinases
α-SMA: α-smooth muscle actin
