# Supplementary material for: Network pharmacology and experimental verification to decode the action of Qing Fei Hua Xian Decotion against pulmonary fibrosis
Source: PLoS One. 2024 Jun 24;19(6):e0305903. doi: 10.1371/journal.pone.0305903 (PMC11195996; doi:10.1371/journal.pone.0305903)
Supplement: S1 Table — (DOC) [file pone.0305903.s001.doc]

**S1 Table. Identified active compounds**

| **Mol ID** | **Molecule name** | **OB(%)** | | **DL** | **Herbs** | **CAS number** |
| --- | --- | --- | --- | --- | --- | --- |
| MOL000006 | luteolin | 36.16 | 0.25 | | MH/TZS/ZS | 491-70-3 |
| MOL000098 | quercetin | 46.43 | 0.28 | | HQ/MH/TLZ/GC | 73123-10-1 |
| MOL000211 | Mairin | 55.38 | 0.78 | | HQ/XR/GC | 472-15-1 |
| MOL000239 | Jaranol | 50.83 | 0.29 | | HQ/GC | 3301-49-3 |
| MOL000296 | Hederagenin | 36.91 | 0.75 | | HQ/TLZ | 465-99-6 |
| MOL000354 | isorhamnetin | 49.6 | 0.31 | | HQ/TLZ/GC | 480-19-3 |
| MOL000358 | beta-sitosterol | 36.91 | 0.75 | | DG/MH/BX/TZS/TLZ/CS/ZBM | 83-46-5 |
| MOL000359 | sitosterol | 36.91 | 0.75 | | XR/CS/GC | 83-46-5 |
| MOL000371 | 3,9-di-O-methylnissolin | 53.74 | 0.48 | | HQ | N/A |
| MOL000378 | 7-O-methylisomucronulatol | 74.69 | 0.3 | | HQ | N/A |
| MOL000387 | Bifendate | 31.1 | 0.67 | | HQ | 73536-69-3 |
| MOL000392 | formononetin | 69.67 | 0.21 | | HQ/GC | 485-72-3 |
| MOL000422 | kaempferol | 41.88 | 0.24 | | HQ/MH/TLZ/GC | 520-18-3 |
| MOL000438 | (3R)-3-(2-hydroxy-3,4-dimethoxyphenyl)chroman-7-ol | 67.67 | 0.26 | | HQ | 64474-51-7 |
| MOL000449 | Stigmasterol | 43.83 | 0.76 | | DG/MH/XR/BX/CS | 83-48-7 |
| MOL000492 | (+)-catechin | 54.83 | 0.24 | | MH/XR/CS | 154-23-4 |
| MOL000497 | Licochalcone a | 40.79 | 0.29 | | GC | 58749-22-7 |
| MOL000500 | Vestitol | 74.66 | 0.21 | | GC | 20879-05-4 |
| MOL000519 | coniferin | 31.11 | 0.32 | | BX | 109664-02-0 |
| MOL001002 | ellagic acid | 43.06 | 0.43 | | CS | 476-66-4 |
| MOL001004 | pelargonidin | 37.99 | 0.21 | | ZBM | 134-04-3 |
| MOL001494 | Mandenol | 42 | 0.19 | | MH/GL | 544-35-4 |
| MOL001506 | Supraene | 33.55 | 0.42 | | MH/TZS | 111-02-4 |
| MOL001689 | acacetin | 34.97 | 0.24 | | TZS | 480-44-4 |
| MOL001755 | 24-Ethylcholest-4-en-3-one | 36.08 | 0.76 | | MH/BX | 67392-96-5 |
| MOL001798 | neohesperidin_qt | 71.17 | 0.27 | | ZS | 13241-33-3 |
| MOL001918 | paeoniflorgenone | 87.59 | 0.37 | | CS | 80454-42-8 |
| MOL001924 | paeoniflorin | 53.87 | 0.79 | | CS | 23180-57-6 |
| MOL001941 | Ammidin | 34.55 | 0.22 | | ZS | 482-44-0 |
| MOL002032 | DNOP | 40.59 | 0.4 | | BL | 117-84-0 |
| MOL002211 | 11,14-eicosadienoic acid | 39.99 | 0.2 | | XR/TLZ | 2091-39-6 |
| MOL002311 | Glycyrol | 90.78 | 0.67 | | XR/GC | 23013-84-5 |
| MOL002372 | (6Z,10E,14E,18E)-2,6,10,15,19,23-hexamethyltetracosa-2,6,10,14,18,22-hexaene | 33.55 | 0.42 | | XR/BL | 7683-64-9 |
| MOL002565 | Medicarpin | 49.22 | 0.34 | | GC | 32383-76-9 |
| MOL002670 | Cavidine | 35.64 | 0.81 | | BX | 32728-75-9 |
| MOL002714 | baicalein | 33.52 | 0.21 | | BX/CS | 491-67-8 |
| MOL002823 | Herbacetin | 36.07 | 0.27 | | MH | 527-95-7 |
| MOL002881 | Diosmetin | 31.14 | 0.27 | | MH/GL | 520-34-3 |
| MOL002914 | Eriodyctiol (flavanone) | 41.35 | 0.24 | | ZS | 4049-38-1 |
| MOL003896 | 7-Methoxy-2-methyl isoflavone | 42.56 | 0.2 | | GC | 19725-44-1 |
| MOL003906 | K-STROPHANTHOSIDE_qt | 30.8 | 0.78 | | TLZ | 33279-57-1 |
| MOL003907 | erysimoside | 65.45 | 0.23 | | TLZ | 7082-34-0 |
| MOL004328 | naringenin | 59.29 | 0.21 | | MH/ZS/GC | 153-18-4 |
| MOL004355 | Spinasterol | 42.98 | 0.76 | | XR/GL/CS | 481-18-5 |
| MOL004446 | 6-Methoxyl-2-acetyl-3-methyl-1,4-naphthoquinone-8-O-beta-D-glucopyranoside | 33.31 | 0.57 | | ZBM | N/A |
| MOL004450 | Chaksine | 65.63 | 0.66 | | ZBM | 486-53-3 |
| MOL004576 | taxifolin | 57.84 | 0.27 | | MH | 480-18-2 |
| MOL004798 | delphinidin | 40.63 | 0.28 | | MH | 528-53-0 |
| MOL004828 | Glepidotin A | 44.72 | 0.35 | | GC | 42193-83-9 |
| MOL004835 | Glypallichalcone | 61.6 | 0.19 | | GC | 146763-58-8 |
| MOL004841 | Licochalcone B | 76.76 | 0.19 | | XR/GC | 58749-23-8 |
| MOL004848 | licochalcone G | 49.25 | 0.32 | | GC | N/A |
| MOL004891 | shinpterocarpin | 80.3 | 0.73 | | GC | 157414-04-5 |
| MOL004903 | liquiritin | 65.69 | 0.74 | | XR/GC | 31564-20-2 |
| MOL004908 | Glabridin | 53.25 | 0.47 | | XR/GC | 59870-68-7 |
| MOL004941 | (2R)-7-hydroxy-2-(4-hydroxyphenyl)chroman-4-one | 71.12 | 0.18 | | GC | 578-86-9 |
| MOL004985 | icos-5-enoic acid | 30.7 | 0.2 | | GC | N/A |
| MOL005007 | Glyasperins M | 72.67 | 0.59 | | GC | N/A |
| MOL005530 | Hydroxygenkwanin | 36.47 | 0.27 | | GL | 6980-25-2 |
| MOL005573 | Genkwanin | 37.13 | 0.24 | | MH | 437-64-9 |
| MOL005828 | nobiletin | 61.67 | 0.52 | | ZS | 478-01-3 |
| MOL006554 | Taraxerol | 38.4 | 0.77 | | TZS | 22076-46-6 |
| MOL006967 | beta-D-Ribofuranoside, xanthine-9 | 44.72 | 0.21 | | BX | 5968-90-1 |
| MOL006992 | (2R,3R)-4-methoxyl-distylin | 59.98 | 0.3 | | CS | N/A |
| MOL007171 | 5-dehydrokarounidiol | 30.23 | 0.77 | | GL | N/A |
| MOL007207 | Machiline | 79.64 | 0.24 | | XR | 2196-60-3 |
| MOL007879 | Tetramethoxyluteolin | 43.68 | 0.37 | | ZS | 855-97-0 |
| MOL010482 | WLN: 6OVR BVO6 | 43.74 | 0.24 | | BL | 84-75-3 |
| MOL010485 | EPA | 45.66 | 0.21 | | BL | 10417-94-4 |
| MOL010489 | Resivit | 30.84 | 0.27 | | MH/BL | 480-17-1 |
| MOL010788 | leucopelargonidin | 57.97 | 0.24 | | MH | 520-17-2 |
| MOL010921 | estrone | 53.56 | 0.32 | | XR | 53-16-7 |
| MOL012922 | l-SPD | 87.35 | 0.54 | | XR | 16562-13-3 |
| MOL013277 | Isosinensetin | 51.15 | 0.44 | | ZS | N/A |
| MOL013279 | 5,7,4'-Trimethylapigenin | 39.83 | 0.3 | | ZS | 5631-70-9 |
| MOL013430 | Prangenin | 43.6 | 0.29 | | ZS | 2880-49-1 |
| MOL013433 | prangenin hydrate | 72.63 | 0.29 | | ZS | 31575-93-6 |
| MOL013436 | isoponcimarin | 63.28 | 0.31 | | ZS | 59176-65-7 |
| MOL013437 | 6-Methoxy aurapten | 31.24 | 0.3 | | ZS | 28587-43-1 |
| - | Mannan | - | - | | SGL | 9036-88-8 |
| - | galactan | - | - | | SGL | 39300-87-3 |
| - | Xylan | - | - | | SGL | 9014-63-5 |
| - | Cellulose | - | - | | SGL | 9004-34-6 |

HQ Huangqi(Astragalus Membranaceus), DG Danggui(Angelica Sinensis), MH Mahuang(Ephedra), XR Xingren(Bitter Almonds), BX Banxia(Pinellia), GL Gualou(Trichosanthes Kirilowii Maxim), TZS Taizishen(Radix Pseudostellariae), TLZ Tinglizi(Semen Lepidii), CS Chishao(Radix Paeoniae Rubra), BL Binglang(Betel Nut), ZS Zhishi(Immature Bitter Orange), ZBM Zhebeimu(Thunberg Fritillary Bulb), GC Gancao(Liquorice)，SGL Sigualuo(Loofah Sponge)
