# Supplementary material for: Network pharmacology and experimental verification to decode the action of Qing Fei Hua Xian Decotion against pulmonary fibrosis
Source: PLoS One. 2024 Jun 24;19(6):e0305903. doi: 10.1371/journal.pone.0305903 (PMC11195996; doi:10.1371/journal.pone.0305903)
Supplement: S1 Raw image — (PDF) [file pone.0305903.s003.pdf]

Fig 8A

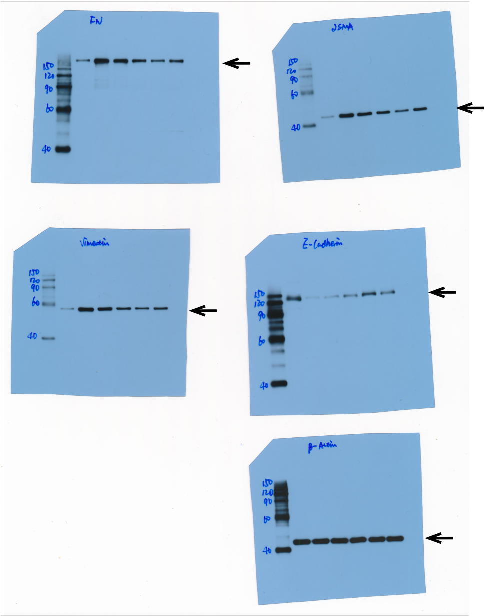

Fig 9A

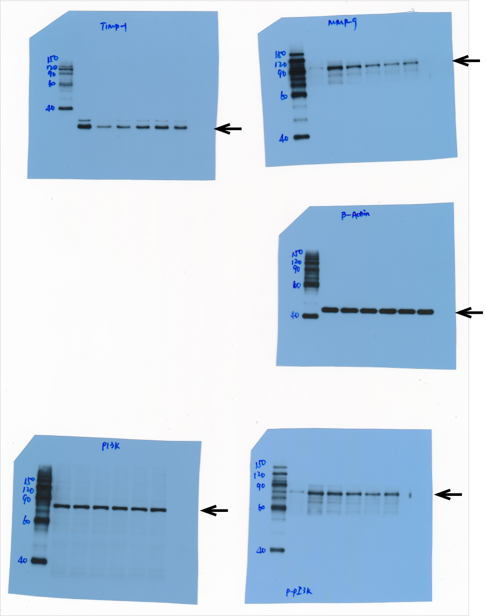

Fig 9B

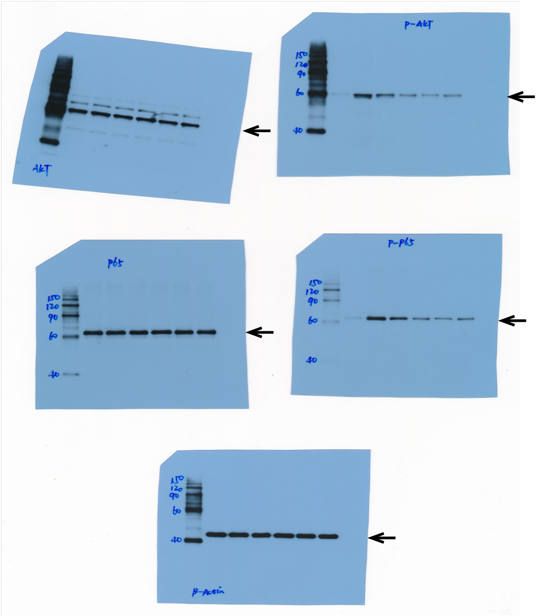

Fig 9C

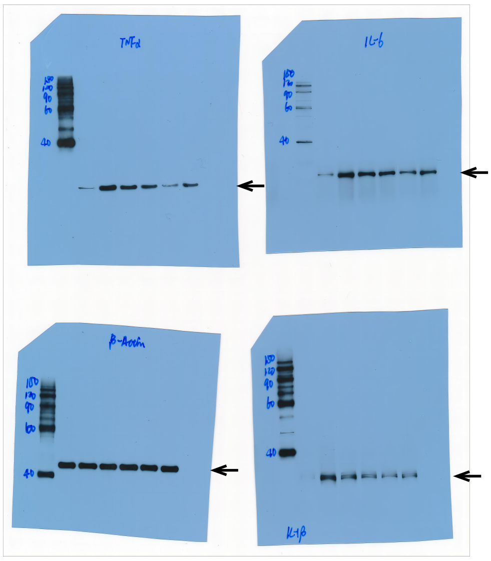

Fig 10A

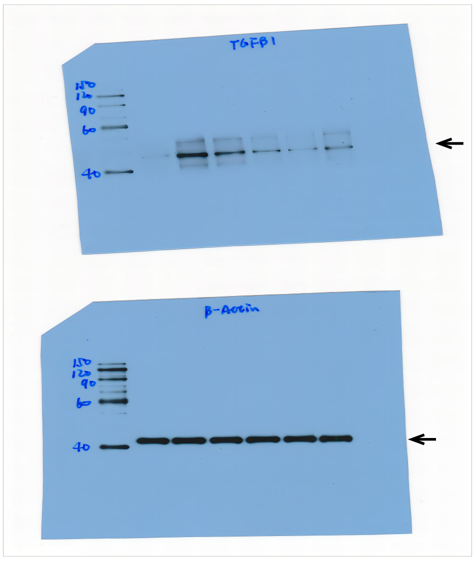

Fig 10B & 10C

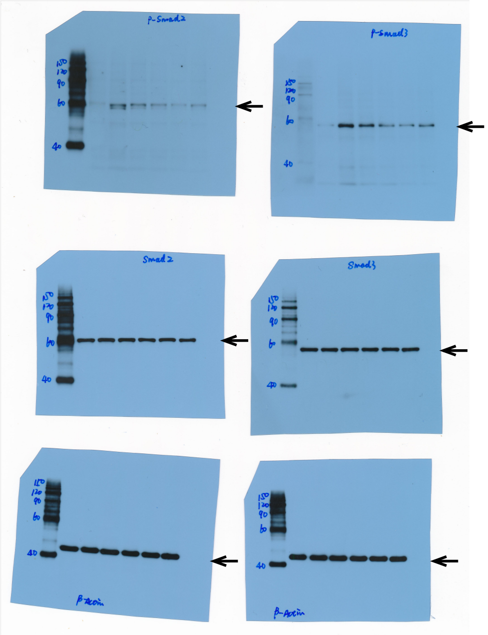

Loading order: (left) marker, control, model, QFHXD-L, QFHXD-M, QFHXD-H, prednisone
